# The transitioning experiences of internationally-educated nurses into a Canadian health care system: A focused ethnography

**DOI:** 10.1186/1472-6955-10-14

**Published:** 2011-06-21

**Authors:** Gina MA Higginbottom

**Affiliations:** 1 Faculty of Nursing, University of Alberta, Edmonton, Alberta, Canada

## Abstract

**Background:**

Beyond well-documented credentialing issues, internationally-educated nurses (IENs) may need considerable support in transitioning into new social and health care environments. This study was undertaken to gain an understanding of transitioning experiences of IENs upon relocation to Canada, while creating policy and practice recommendations applicable globally for improving the quality of transitioning and the retention of IENs.

**Methods:**

A focused ethnography of newly-recruited IENs was conducted, using individual semi-structured interviews at both one-to-three months (Phase 1) and nine-to-twelve months post-relocation (Phase 2). A purposive sample of IENs was recruited during their orientation at a local college, to a health authority within western Canada which had recruited them for employment throughout the region. The interviews were recorded and transcribed, and data was managed using qualitative analytical software. Data analysis was informed by Roper and Shapira's framework for focused ethnography.

**Results:**

Twenty three IENs consented to participate in 31 interviews. All IENs which indicated interest during their orientation sessions consented to the interviews, yet 14 did not complete the Phase 2 interview due to reorganization of health services and relocation. The ethno-culturally diverse group had an average age of 36.4 years, were primarily educated to first degree level or higher, and were largely (under) employed as "Graduate Nurses". Many IENs reported negative experiences related to their work contract and overall support upon arrival. There were striking differences in nursing practice and some experiences of perceived discrimination. The primary area of discontentment was the apparent communication breakdown at the recruitment stage with subsequent discrepancy in expected professional role and financial reimbursement.

**Conclusions:**

Explicit and clear communication is needed between employers and recruitment agencies to avoid employment contract misunderstandings and to enable clear interpretation of the credentialing processes. Pre-arrival orientation of IENs including health care communications should be encouraged and supported by the recruiting institution. Moreover, employers should provide more structured and comprehensive workplace orientation to IENs with consistent preceptorship. Similar to findings of many other studies, diversity should be valued and incorporated into the professional culture by nurse managers.

## Background

The International Council of Nurses [1] has predicted an unprecedented global shortage of both clinical and academic nurses. Indeed, high income nations such as Australia, Canada, the United States and the United Kingdom have for several decades redressed deficits in the nursing workforce via recruitment of internationally educated (IENs) and trained nurses [2-4]. Strategies of this nature are fraught with ethical dilemmas [5]. In summary, the mass movement and relocation of a highly skilled workforce has consequences for both the country of origin of the recruited nurses and the host community (both professional and lay). Most notably for the country of origin, are the profound effects on health care provision (in terms of nurse-to-patient ratios) that can occur when developing or low-income countries as a development strategy train large numbers of nurses who subsequently migrate [6].

Since health care systems are configured and evolve in relation to socio-economic, political, and cultural circumstances, there may be wide variation in the context of health care delivery and in the education and training of health professionals, including nurses [7]. These variations may mean that the professional competencies, including ethno-cultural components, nurses achieve during core education may or may not be directly transferable to another health care context. Historically, issues faced when acquiring credentialing are the major barriers for IENs and other foreign-trained professionals hoping to obtain meaningful employment [8-10]. Indeed, the main challenge many IENs face is finding that their qualifications do not meet the standards in the destination country [10,12-16]. While bridging/retraining/adaptation programs are available in some countries, these at present generally lack standardization and are often of suboptimal content. An often delayed licensure and registration promotes the "deskilling process" [14], consequentially IENs are forced to work lower level jobs and are retained within these positions for extended periods due to inconsistent training policies and both overt and subtle forms of discrimination [11,13,17]. Beyond limiting the potential of IENs to reach their full potential through competency recognition and promotion, deskilling and discriminatory practices may foster feelings of invisibility and marginalization with long-term effects including reduced self-esteem, confidence and possibly psychological health and well-being.

In addition to the credentialing issues that have been well-documented[10,18,19], new immigrant nurses may need considerable support in transitioning into new social and health care environments. Cultural displacement is frequently described and largely encompasses: language and communication barriers, feelings of being an "outsider", and differences in nursing practice. Nursing is a profession that highly depends on clear and accurate communication to ensure patient safety and treatment/care outcomes. Several international studies have reported that language is the most significant barrier to successfully gaining employment as well as integrating within the foreign country [12,15,20-22]. One study of overseas nurses in Australia found that nurses fluent in English as their first language were more likely to secure employment in their preferred specialty [23]. A strong command of the language may also assist with cultivation of friendships and support from peers, both of which are predictors of assimilation. The psychosocial terms of cultural dissonance (characterized by extreme anxiety) and disillusionment have been used to describe the experiences of some IENs [7,22]. Others whom have come from culturally-similar countries may fare better, since IENs are often expected to adapt to the host culture in a limited timeframe and without performance gaps [24].

Differences within the host countries nursing practice have been recognized as difficult during and beyond transition for many IENs. Limited knowledge of the health care system policies and practices (around confidentiality, documentation, patient care accountabilities, and interdisciplinary roles), tax laws, and social security rules among others can make the transition very difficult for nurses [20,22,25]. Differences in autonomy in nursing practice are noted by several authors, who have shown this can be country-specific and may cause a shift in professional identity [14,25]. Ignorance of the health care systems technologies, and thus the overall practice of information management, has also been a finding which may be long-lasting for some IENs [26].

While the above discussion highlights research findings on IENs' experiences during transition in some countries, research in the Canadian context has been scarce, performed within its Eastern provinces,[10,27-29] and largely focused on credentialing issues [9,10,12,27-31]. Studies suggest that IENs articulate the confusion and subsequent disillusionment when told of their need for Canadian licensure [7,10]. Many IENs have faced financial hardships as they endeavoured to obtain the necessary documentation of their education for entry into Canadian upgrading programs [6]. Moreover, one study noted that there is no standard source in Canada where applicants may obtain information on educational requirements prior to entry into Canada [32]. There are also limited support structures in place using a second language to aid IENs in gaining examination skills [29,33]. In summary, IENs are experiencing frustration and disappointment with some leaving Canada prematurely which may create a financial drain on the host community and the individual nurse [19,27,34]. Recently in Canada, strategies have been established to enhance integration of internationally qualified health professionals into the Canadian health care system. At a federal level (but with key provincial contacts) the Pan-Canadian Framework for the Assessment and Recognition of Foreign Qualifications, as conceptualized to aid recognition of foreign credentials and smooth the transition of individuals from key occupations including nursing, was implementation December 2010 [35]. The strategy maps out a one-year time frame, from pre-migration to active participation in the workplace, during which assessment, recognition, and partial or non-recognition of credentials occurs.

During 2008-2009, a health authority in a western province in Canada undertook recruitment drives in Australia, India, the United Kingdom and the Philippines to address regional nursing workforce deficits, resulting in 800 contingent job offers. The first cohort of ten nurses arrived in early 2008, with the remaining nurses planned for arrival during the subsequent 12 to 18 months. This timeframe was therefore a critical juncture at which to conduct this research and harness vital data of relevance to federal and provincial decision makers.

The aim of this study was to gain an understanding of the transitioning experiences of IENs upon relocation to Canada, with the additional goal of creating some national and/or provincial policy and practice recommendations for improving the quality of their transition and their retention. Our primary research question was *How do IENs transition into the Canadian health care system? *In order to gain a comprehensive understanding of this question a number of subsidiary research questions were proposed, these being grounded in the findings of international research studies exploring the experience of IENs [2,6,36,37]:

1. What are the motivations of IENs for relocation to western Canada?

2. What are the expectations of IENs in terms of their professional role in the Canadian context?

3. What are IENs' experiences of recruitment, reception and support on arrival?

4. How does their working life differ from that in the country of origin?

5. Have the IENs experienced racism and/or discrimination since arrival?

6. How is living in Canada experienced by IENs?

7. What strategies have IENs employed to overcome obstacles and barriers?

This focus on the Canadian context will not only assist those regulating, recruiting and training/supervising IENs in Canada, but will also provide some additional insights for regulatory bodies and health care managers and commissioners in other countries when recruiting IENs and implementing bridging/adaptation programs which will ensure successful transition of their IENs and thus their provision of health care.

## Methods

### Design

This qualitative study was designed utilizing the principles associated with a focused ethnography [38], and an approach located within the interpretive paradigm which is appropriate for an exploratory descriptive study of this nature and a focus on a sub-culture or group such as the IENs in this study [39]. A phenomenological approach was rejected in favour of focused ethnography because of the latter's relevance to the health care context as evidenced in growth of this genre of research in health-care [40] and its congruence with the research questions posed in this study. Focused ethnographies are well-suited for health research answering specific questions (formulated prior to conducting the research) and are characterized by:

▪ Conceptual orientation of a single researcher;

▪ The focus on a discrete community or organisation or social phenomena;

▪ Being problem-focused and context-specific;

▪ Involvement of a limited number of participants;

▪ Participants who usually hold specific knowledge;

▪ Episodic participation observation (in some conceptualizations but may be omitted); and

▪ Their use for development in health services [40].

Focused ethnographies may have a limited number of data collection strategies and participant observation is not a requirement [41]. Because of the diverse employment patterns of the IENs participating in this study, it was not feasible to facilitate participant observation although in future studies this might be desirable to afford greater insight into interactions of the IENs and their co-workers or managers.

Semi-structured interviews with one group of IENs were conducted at both one to three months post-relocation (Phase 1; whilst the IENs were in the initial stages of transition), and at nine to twelve months post-relocation (Phase 2; when the IENs were likely becoming more familiar with the new cultural context and health care delivery systems). Key-stakeholders (e.g. representatives of the health authority and regulatory body) were also interviewed (Phase 3) although this data has not yet been analyzed and will be reported in a subsequent publication.

### Sampling and Recruitment

Purposive sampling was undertaken, since all of the participants would essentially come from, in ethnographic terms, a similar subculture (of IENs) and would be having specific experiences of interest to the researcher [42]. A sample size of 30 was estimated to achieve data saturation and maximum phenomena variation in the sample with regard to age, gender, ethnicity, and dependant status. Some participants were visible minorities and some were not, however this dimension is important in terms of eliciting views on perceived discrimination by those individuals who are not similar in phenotype to the majority group (Table 1).

**Table 1:** Biographical Details of IENs in Study

Code #	M/F	Age (yr)	Self-assigned ethnicity	Spoken language	Qualification	Place of training	Other countries where worked	Entry in Nursing	Current position	Previous position
1	M	38	Filipino	Filipino	BSc in Nursing	Philippines	Saudi & U.K.	1993	GN	RN
2	F	50	Filipino	Filipino	Masters in Public Health	Philippines	Saudi & U.K.	1980	NA	Tutor/UN Co-ordinator
3	F	48	Filipino	Filipino	Masters in Nursing	Philippines	N/A	1981	NA	Admin
4	F	43	New Zealander	English	BSc in Nursing	New Zealand	N/A	2000	GN	RN
5	F	35	Filipino	Filipino	BSc in Nursing	Philippines	Saudi & U.K.	1994	GN	RN
6	M	41	New Zealander	English	BSc in Nursing	New Zealand	Tonga & Pakistan	1989	GN	Staff Nurse
7	F	36	Filipino	Filipino	BSc in Nursing	Philippines	New Zealand	1996	GN	RN
8	F	30	Black	English	BSc in Nursing	The Caribbean	USA	2004	GN	RN
9	F	41	Black	English	BSc in Nursing	The Caribbean	U.K.	2001	GN	Staff Nurse
10	M	26	Australian European	English	BSc in Nursing	Australia	U.K.	2004	GN	RN
11	F	30	Australian European	English	Masters in Midwifery	Australia	U.K.	2003	GN	RN & RM
12	F	46	British	English	Diploma in Nursing	UK New Zealand	None	2002	GN	RN
13	F	39	Filipino	Filipino	BSc in Nursing	Philippines	None	1991	LPN	Staff Nurse
14	M	43	Black African	Akan	MA, BA (Hons), RMN	United Kingdom	U.K.	1992	PN	Care Coordinator
15	M	25	Filipino	Tagalog	Bachelors of Nursing	Philippines	Philippines	2005	LPN	Staff Nurse II
16	M	43	British	English	Diploma in Adult Nursing	M: UK	United Kingdom	M: 2001	C.G.N. (Cert. Graduate Nursing)	Clinical Nurse Manager, RN (Male)
17	F	46	British	English	Diploma in Adult Nursing	Unknown	New Zealand	1995	C.G.N. (Cert. Graduate Nursing)	Clinical Nurse Specialist, RN
18	F	N/A	Caribbean	English	BSc & Diploma	United Kingdom	Not Given	2004	C.G.N. (Cert. Graduate Nursing)	R.P.N. (Reg. Psych. Nurse)
19	M	25	Ilonggo	Tagalog	BSc in Nursing	Philippines	None	2005	LPN	Clinical Instructor
20	F	23	Filipino	Tagalog	RN, BSN, NCLEX-RN	Philippines	None	2006	LPN	Staff Nurse
21	M	31	Asian	Taglog	BSc Nursing, BSC in Med Tech	Philippines	None	2005	LPN	Staff Nurse
22	F	27	Filipino	Tagalog	RN, BSs Nursing	Philippines	None	2004	LPN	Charge Nurse
23	F	35	Filipino	Filipino	BSc in Nursing	Philippines	None	1995	LPN	RN level 3

The participants were recruited during their week-long orientation to the health authority held at a local college. The principal investigator, herself an IEN although not practicing clinically, made arrangements with the health authority to personally present the study at the commencement of these orientations, leaving potential participants with information sheets and reply slips. The investigator introduced herself and provided some background of her research and qualifications. Some general background of the study was provided and the aim of the study was fully articulated, yet without any reference to her personal assumptions or background findings from other studies. The responses demonstrating interest were subsequently collected from a mail box and contact was then made with the IEN by the research team. Additionally, a 'snowballing' strategy was used by asking recruited participants to pass along information about our study to their IEN friends and/or colleagues. Some participants were also recruited through the local association for Filipino nurses.

### Data Collection

In the spring of 2009, 23 IENs participated in 31 interviews - 22 during Phase 1, where one couple interviewed together (at their request), and 9 in Phase 2. The majority of the interviews were performed by the principal investigator and one graduate research assistant (also a female IEN); although a few were performed by one of two research assistants who were not IENs but both of a visible minority. The principal investigator has extensive training and experience with qualitative interviewing, particularly with minority populations, and provided training with supervision to the other interviewers. The originally planned sample of 30 IENs, and indeed the majority of Phase 2 interviews, could not be achieved largely due to reconfiguration of the health authority in the province, with associated radical financial cutbacks leaving the recently-recruited IENs employed on a one-year contract without the opportunity to renew their contracts. Ultimately, although many of the IENs had two-year work Visas many returned early to their country of origin.

Contact was made by the study investigator or research assistant by telephone to all interested IENs and Phase 1 interviews were scheduled to take place at the IEN's place of residence or at the University in a private interviewing room or workplace, according to the IEN's preference. Prior to the interviews, the study procedures were explained in detail, confidentiality was assured, and full informed consent was provided by all participants. After this point, detailed demographic/biographical information using self-assigned ethnicity and nationality was collected from participants in order to construct a profile of the IENs. The interviews then commenced, guided by a topic-guide and recorded by a digital recorder.

The investigator and graduate research assistant developed the first semi-structured interview/topic guide and solicited comments from an advisory group of key stakeholders within the health authority and regulatory body for registered nurses within the province. The topic guide was then piloted with the first four study participants and analyses of the pilot data enabled further iteration and refinements to the guide for the remaining 18 interviews. The guide consisted of overarching topic headings related to our research questions with specific questions within topic each to help serve as prompts during the interview if the IEN did not discuss the topic in enough depth to gather comprehensive data. Field notes were written after each interview to allow for later reflection during team progress and data analysis meetings. Meanwhile, the advisory group reconvened to construct the second interview guide taking into account emergent issues and the need to provide a temporal comparative dimension.

### Ethical considerations

Ethical approval of the protocol including recruitment methods was obtained from the Research Ethics Board for the investigator's institution and institutions within the governance of the health authority, and informed consent was provided by each study participant.

### Data Management and Analysis

The audio-recordings were transcribed verbatim by a professional transcriptionist and verified by the respective interviewer. Data was then stored, managed, classified and ordered with the aid of NVivo 8 (QSR International, Victoria, Australia) qualitative data analysis software. We applied Roper and Shapira's [43] framework for the analysis of ethnographic data which incorporates: 1) coding for descriptive labels, 2) sorting to identify patterns, 3) identification of outliers or negative cases, 4) generalizing constructs and theories, and 5) memoing to note personal reflection and insights. Codes were assigned, by the principal investigator and graduate research assistant, to reflect categories in relation to both the specific research questions and to reflect emerging or other persistent concepts which were not anticipated. Reflective team meetings were also convened at critical junctures during the analysis period. In this study we also conducted an in-depth and detailed analysis to enable comparisons to be made between the first and second interviews to establish any changes in perceptions or experiences regarding their transitioning.

## Results

The quantitative data profiling the IENs were tabulated (Table 1) and the qualitative data was organized and classified into seven key themes including (the dominant Phase which generated the data is indicated):

• Motivation and Decision to Relocate (Phase 1)

• Expectations versus Actual Personal Experiences of Recruitment, Reception, Salary & Support on Arrival (Phase 1)

• The Health Care System Nursing Work Environment (Phase 1 & 2)

• Discrimination in the Professional Lives of IENs (Phase 2)

• Qualifying as a Registered Nurse (Phases 1 & 2)

• Life Beyond the Nursing Setting (Phase 1 & 2)

• Strategies IENs Learned to Overcome Challenges (Phase 2)

### Profile of the Participating IENs

The collected details present an interesting profile of the participating IENs (Table 1). The nurses had diverse ethno-cultural origins in the Philippines, Australia, New Zealand, England, Scotland, Barbados and Ghana. Time from completion of their nursing qualifications ranged from 4-19 years and their average age was 36.4 years. Almost all were educated to first degree level with some holding master's degrees. No participant had acquired a Registered Nurse (RN) qualification within Canada and they were largely employed as graduate nurses with a small number being employed as nursing aides.

### Motivation and Decision to Relocate

This study reveals that the motivations of the IENs for migrating into Canada were wide-ranging. These can be broadly categorized as personal and professional motivations. Personal motivations included the search for better economic opportunities and a generally better life (particularly for their families), for a place different from the IEN's home country, or the desire to join relatives already in Canada, among others. Several IENs desired financial gains to help fulfill family financial needs in their home country. Other personal motivations included adventure and the experience of a new ethno-cultural context. Professionally, the IENs relocated to western Canada largely due to their desire to learn new skills, to widen their work experience, or to access further education. Some stated that they were leaving unfavourable former working conditions:

*"What brought me to Canada is [that] I always like to experience a different climate of nursing."***(Participant 9)**

Findings also demonstrate that the motivation to relocate to Canada for better economic opportunities and improvements in quality of life was mediated by the IEN's country of origin. Only those IENs who migrated originally from developing countries or low income nation states referred to the above as the main motivation for their migration. On the other hand, those from the more affluent, high income countries were motivated primarily by the desire to travel, be away from home, or to experience Canada. Indeed, some of these IENs perceived Canada to have more affordable living standards. The following quotations, from Filipino and European participants respectively, exemplify this:

*"Honestly we are earning here A LOT compared to my home country. So that's one of the main reasons because I really want to support my family back home.... Well, a staff nurse in the Philippines is earning 6000 pesos[approx $130 CD] and if here, well I could say that our salary here for one month could be the equivalent of one year's salary in the Philippines."***(Participant 18)**

*"We worked in the United Kingdom a few years ago, and we just wanted to have another travel work experience. We like the outdoors and things like that so we wanted to have a look at the mountains and animals, and I like fishing and things like that so. Really, other than that, there was no real intention." *(**Participant 10)**

### Expectations versus Actual Personal Experiences of Recruitment, Reception, Salary & Support on Arrival

Whereas personal factors were the main motivator for the IENs' desire to pursue a nursing career in Canada, their expectations were by and large related to their careers. When asked about what she thought of Canada prior to relocation, one participant noted:

*"I think Canada is a better place because I can learn more here especially with my profession. In the Philippines we are lacking of supplies. I must admit that we are not doing good in procedure or something because we are lacking of supplies. We let [patients'] relatives buy supplies. Let's say we are going to do one dressing. We will ask relatives to buy supplies, like gauze. And even syringes...."***(Participant 13).**

Some of the IENs' expectations were met while in other cases reality fell short of expectations. In fact, IENs' negative experiences of recruitment, reception and support on arrival stemmed largely from these unmet expectations. A great deal of IENs' negative experiences related to absence of appropriate or sufficient supports on arrival. Some perceived their experiences upon arrival to Canada to be positive, appreciating collection at the airport, arranged accommodation for a period of six-weeks, and the provision of necessary information, while others had negative experiences including failure to receive arranged transportation, difficulties navigating the process of obtaining a Social Insurance Number (essential for payment of salary), and displeasure with the dormitory-type accommodations (offering the bare necessities, a shared kitchen, bathroom and lounge). The IENs also repeatedly complained about the geographical location of their accommodations which were thought to be assigned without regard to proximity to their work location:

*"My first accommodation was in W. That is near X Mall. That's at the back of Y (Hospital). I wasn't pleased at all when they put me there and I will work here. So during the briefing I told them my concerns that we came here in Canada because of these benefits or this offer - that $5000 relocation package, free accommodation and stuff like that. But it seems that your accommodation is quite inconvenient for the people, you know, like me who will work in Z Hospital but I live in W [geographically distant]." *(**Participant 7).**

As related to the IENs' work contracts with and financial reimbursement from the health authority, significant discontent was expressed by many IENs particularly with regard to their expectation of working in their area of specialty. For example, several IENs thought that prior to relocation they were not given sufficient or accurate information about their job positions or their job tasks in Canada. However, many were recruited by agencies in the country of origin rather than directly by the employing authority, therefore it is difficult to establish whether accurate information was provided which reflected the actual employer perspectives. Some IENs commented that their contracts were changed just prior to their move:

*"We were told that ... you will always work in your area of specialty which has turned out to not be true. They guaranteed that you would always, if you are specialized in an area, then that is where you go. ... they kept changing what they wanted to do with me every five minutes. And then two weeks before I left to come over here, like I was supposed to go to Emergency (I'm trained in Emergency and that is all I have ever done) they say 'oh, there is no positions anymore. You have to work on a medical ward.'" *(**Participant 10)**

Others thought that after their arrival the nurse manager in charge of their placements failed to discuss with them details of their specialty and instead placed them in different areas. The following comment is reflective of the views of many of the participants:

"To me it was more suicidal than anything... the contract was that um, temporary full-time for one year... *almost all Internationally Educated Nurses who came, who didn't really understand what that entailed until we got here... given all those years of experiences of working in the NHS no one would have swapped that for a temporary one year position...then because they used an agency, the people that they employed at the agency themselves didn't really understand that." ***(Participant 14)**

Many IENs also had difficulty reconciling the fact that they were recruited as RNs by the health authority but the regulatory body did not allow them to practice as RNs (see section on Qualifying as a Registered Nurse). Moreover, because of employment at graduate nurse level, not RN, many IENs stated that their actual salary entitlements were lower than those included in their signed contract. An additional financial challenge faced by many IENs were the long-wait times (up to eight weeks was claimed) for receiving their reimbursement of expenses.

When asked about their experiences of reception once starting work, many IENs were surprised that they were not introduced to their co-workers on their first day as might be considered courteous. Moreover, although their co-workers of similar ethno-cultural background were friendly, fellow Canadian workers were typically viewed as friendly in a capricious manner. One IEN described a substantial incongruity between previous experiences in another country where staff members were informed in advance of newly recruited staff and where introductions on arrival were commonplace.

### The Health Care System Nursing Work Environment

When asked to share their general perspectives on the working within the provincial health care system, the overwhelming sentiment towards the system was negative. Only occasionally did the IENs feel positive about the system. In one such case an IEN felt that the system was generally supportive of IENs.

*"What I appreciate with them [the health authority] is that they just don't throw you into the job. They orientate you as best as possible. And even here, they don't just say 'okay, you're in theatre today.' They try to sensitive you to the things that you're supposed to know except for those little bit flaws that I had just say." ***(Participant 9).**

While many of the IENs were enrolled in a six-week long orientation/retraining session, many felt that there were several negative aspects of its implementation and content. Several felt that there was no new information provided to them and that there was an overall lack of consistency within the program particularly with respect to continuity of preceptorship. Others were left without assignment of a preceptor and were trained at a task by watching a staff nurse perform it once or twice.

Some IENs felt disillusioned after relocation to Canada, having realized that the system was not as comparatively advanced as they had expected:

*"I thought that the [Canadian] health care system would be really advanced. Like I thought it would make Australia's health care system look really old-fashioned? But I don't think that's true. Like that's just what I thought in my head. I thought I would come over here and there would just be all this whiz/bang technology, and all these different practices and I wouldn't understand and I thought I'd have lots and lots to learn. But that hasn't ended up being a really accurate idea that I had." ***(Participant 11).**

Others regretted what they perceived as a belief among Canadian health care decision makers and practitioners that their way is always the right way. The IENs particularly pointed out Canadian co-workers' consistent aversion to change (where the IEN was the change agent), a tendency that some IENs considered self-destructive:

"*I think they are a little bit foolish doing what they're doing. I think they are chopping themselves off at the head by doing it. As an educator in New Zealand ... we had lots of international nurses come through the unit. And I loved the fact that they brought skills to New Zealand that we didn't have in New Zealand. You know, and everybody brought something. Canada's not about that unfortunately, or not so far. It's about their way is the right way. ..." ***(Participant 4).**

While the above findings are in respect of IENs working directly for the health authority, some nurses were recruited to work in privately-owned continuing care facilities with the services of these facilities being contracted by the health authority. Indeed it is this cohort who appeared to experience the most dissatisfaction. The extent to which the health authority was fully aware of the work environments of these nurses is not clear.

*"We were sent there by [the health authority] because they're adult clients that was funded [through the provincial health care plan] so we can work there. They said that we're just going to be added staff. So what we're going to do is assist with the medication administration. But then when we get there, the Health Care Aide is the one who gave us the orientation. She oriented us where the kitchen is. Where the cleaning cart is. So the moment I get home, it was already 11 p.m. and I can't call my supervisor... And the [senior] supervisor told her [junior supervisor] that it is part of our job. That as Community nurses, it is different from working in an acute setting and that we should be enthusiastic about, you know, our job. We should be...we should be ready to do, to do those stuff for the resident, to keep them safe, to have the environment tidy. But after doing it for two weeks or three weeks, I've been repeatedly telling them that no this is not what I'm supposed to do here. I just thought that I would just go home if they don't stop that." ***(Participant 19).**

The IENs we interviewed made observations concerning clinical nursing practice differences between their home countries and Canada. While some IENs stated that nursing remains nursing and patients are patients, some observed major changes to their nursing practice in their home country:

"*My biggest challenge. Like knowing that for example, most women here, way over 90 percent of women here, deliver in stirrups, in the lithotomy position. It has been proven time and time again through research that that is a very bad position for her comfort as well as for tearing. It increases tearing risks of the perineum. And they just, they're just like this is the way we've always done it so we'll just keep doing it. And I'm like, that's that woman's body, that's her life, it's her self-esteem, it's her choice, it's her sexuality, it's her relationship with her partner when she goes home. Not just, it's not just about you and the way you've always done it. It should be about that woman and how many aspects of her life this can negatively impact because she's having an unnecessary third degree tear whereas if you let her deliver in an up-to-date position, she might only get a first degree tear... That's a big challenge for me because I'm very passionate about women being looked after in the best possible way. And then I see its old fashioned here and that really, it's upsetting." ***(Participant 11).**

Some IENs also commented on the many ways and reasons for charting (recording of nursing notes and observations) on the wards. Charting for some required learning to use much more objective language (e.g. measurements) and waiting until the end of shift, rather than using more descriptive language in an immediate fashion. Clinical practice varied in other ways beyond patient care extending into professional (interdisciplinary) relationships.

*"Communications for, between the... disciplines between the doctors and the nurses and the Physios and the Occupational Therapists, there's not a feeling of collaboration. There's a feeling of competition. There's a feeling of, far more solid than I experienced. In New Zealand, everybody appreciates what everybody is doing." ***(Participant 4).**

### Discrimination in the Professional Lives of IENs

IENs also shared perspectives on issues of fairness and equity. Some IENs felt that being assigned tasks that were unrelated to nursing, such as dishwashing and vacuuming was reflective of employer/supervisor discriminatory practices based on country of origin. Others felt that the IEN's country of origin determined whether or not they were able to relocate with their family members. Other perceptions of discrimination or unfair treatment mainly related to credentialing and are addressed in the next section.

Some IENs described their perceptions of discrimination in the form of overt racism:

*"Well, what people will talk about, you know, racism. Racism is a big word. Right? People wouldn't come you know and tell you in your face, straight to that because you know, you are black or this or that, I'm doing this to you. Right? It can be very subtle. Very covert." ***(Participant 14)**

### Qualifying as a Registered Nurse

All of the IENs we interviewed were recruited by the health authority as RNs but once in Canada were required to work in different capacities such as a Graduate Nurse, a Licensed Practical Nurse, or a Nursing Aid. For this reason, although this study was not designed to focus on credentialing we found this issue too persistent and troubling to avoid reporting. The main concerns of the IENs focused on being informed that they were here (Canada) to work as a Licensed Practical Nurse and that they could not apply to take the national Canadian Registered Nurse Exam to become an RN. (Registration of nurses in Canada is a provincial matter, yet each province uses the national board exam as part of their credentialing process. Obtaining a practice permit with the regulatory body related to this study is a three-step process which starts with an eligibility assessment requiring that the applicant is currently registered as an RN in the country where they obtained their education and considering whether their education was of generalist nursing at a baccalaureate level. Evidence of English competency [using one of two examination methods] and various practice competency information is also required with the cost of all translation incurred by the IEN). Many of the IENs were not well informed of the criteria used when appointing IENs to the entry level of nursing (i.e. Graduate Nurse) at which they could expect to commence their employment in the province. Some IENs stated their expectation that the regulatory body would adjust credentialing criteria according to the IEN's country of origin, with their perceived reality to be that there were unclear guidelines on the criteria used to assign IENs to the different nursing levels.

*"But we never expected that it [credentialing process] will be so long. And it's really different. Yeah, one thing that was really, that was really promised us is that being an RN right? They didn't tell us about the SEC, that's the Substantial Equivalent Competency Exam that we have to go through for like 5 days. That's written and oral. And then after that, you, um, they're going to assess us as to how many courses are we going to take. So never, never in our wildest dreams."***(Participant 22).**

To some extent the inconsistencies in professional status on arrival may represent a degree of naivety on the part of the IENs, in that perhaps more research and investigation might have been initiated by them at the point of recruitment. Indeed some IENs acknowledged this as an issue. However, many had received RN designations from multiple countries, the United Kingdom and Saudi Arabia for example, which had similar criteria for nursing qualification, and therefore they assumed they would become an RN on arrival without taking a qualifying exam. One participant's comment reflects the overall responses related to dissatisfaction with what the IENs' perceived to be stringent rules of the regulatory body relating to requirements for practicing nursing in the province.

*"It [regulatory body] has put up lots of barriers along the way and you know, if I may pass another exam and stuff, it [regulatory body] is like, "no, you just need to do this, and do this, and do this." It's incredibly pedantic about qualifications you need and stuff like that. I'm sure it must frighten people away.... The hoops they make you jump through are above and beyond what is necessary. I know they have got to be safe with who comes through here but it's too much." ***(Participant 4).**

The findings demonstrate that many IENs were not informed during recruitment of the different stages and qualifying procedures the provincial regulatory body required before one is able to take the national board exam to become an RN. The IENs often questioned whether the regulatory body and employer credentialing decisions were made on a case-by-case basis or based on existing stereotypes, e.g. country of origin or practice of IENs. Many IENs stated their discontent with their perceived inconsistency with decisions related to which IEN writes which credentialing exam; many thought that only considering the last country and position where they worked, rather than their skills and competency, was short-sighted and unfair. Moreover, the content of the national board examination is essentially bound by Canadian ethno-cultural conventions and many IENs are not fully familiar with these conventions which may have lead to disadvantages.

### Life Beyond the Nursing Setting

When we asked the nurses how they were experiencing living in Canada, responses generally considered the financial, health and education institutions they with their families were required to utilize during their stay. In many cases, the IENs could not afford to bring along immediate family members, yet if they were fortunate to do this, many of their spouses experienced difficulty securing employment in their area of specialty. There was a lack of familiarity with the public school system which IENs having young children encountered. Accessing or obtaining vital services including health services, banking, communications (i.e. phone contract), and acceptable and affordable food, was felt to be challenging. Several IENs experienced delays in obtaining an appointment with a family physician. The cost of living was seen as relatively expensive, especially costs of food and other necessities like housing. Many IENs expressed that many of their negative views were shaped by their being paid lower salaries that they were expecting and the uncertainty of reimbursement for their expenses.

"*So I've come here and I've been kind of told, well you can't, you won't be paid as a Registered Nurse until you sit the exam. In the meantime you'll be a graduate nurse. So you're paid at a graduate nurse level. And then your pay goes up once you sit the exam." ***(Participant 6)**

### Strategies IENs Learned to Overcome Challenges

The participants reported a wide variety of strategies for coping in their environment, sometimes related to family and friend supports, communication, reliance on previous experiences, and previous and gained knowledge and perceptions. Many IENs commented on the role played by family members, whether living in their country of origin, living with them in Canada, or being additional family members with whom they joined in Canada. Many IENs were required to start work early upon their arrival, thus they greatly appreciated assistance of supporting family members in shopping, maintaining the living space (cleaning, cooking etc), or locating a more permanent place of residence. One participant mentioned relying upon family members to help supply documents require for their credentialing. More helpful for many, family members were those with whom to "vent to and relax with" whenever working conditions were not favourable.

"*Our two daughters are coming at Christmas with our new grandson and my mom and dad are coming next year. And our youngest son and his girlfriend are hopefully coming next year". ***(Participant 16)**

"*Yeah, we, and the church we go to it's brilliant because we've made a lot of good friends there and so we socialize with them as well". ***(Participant 12)**

Communicating issues was an important way for some IENs to have their issues known. This was likely more productive for those who had a fully fluent command of English, as it has been noted by many investigators that communication abilities may be associated with better chances of becoming employed in the IEN's area of specialty and more expeditious migration and credentialing processes [23,44].

Gained knowledge and knowledge from past experiences were recognized as importance means to facilitate transitioning. IENs who had previously relocated for nursing employment knew that obstacles would need to be faced, and that having an open-mind to learn "their way" would ease the adjustment of the foreign system. Continual learning was one way the IENs found to enhance their assimilation into the Canadian health care system and social environment. Some thought it okay to "learn and practice the Canadian way of doing things even though they go against 'your' practice and beliefs".

"*You have to fend for yourself. And you do whatever it takes to survive. And in a sense, that's the reason why, as I've said, lots and lots of the staff, over half of them, and some are even in the process of leaving". ***(Participant 16)**

## Discussion

### Strengths and Limitations

The author endeavoured to achieve maximum phenomena variation in the sample. While the sample size was reduced due to financial cut-backs within the newly configured health authority, it is believed that the collected demographic and biographical data does indicate wide variation in regard to age, gender, and ethnicity, and dependant status. Additionally, data saturation was determined to be achieved since many of the IENs responded with similar views and there were few divergent themes. Additionally, the findings in general were consistent with research of other IEN experiences in developed nations [16,45], with the resounding perception that previous experience and skills of the IENs were not appreciated or valued by managers, or considered adequately during credentialing. Apart from data saturation, rigor and robustness in qualitative research is to some extent established via a self-conscious and reflective approach accompanied by an explicit methodological framework. Research team meetings enabled articulation of reflexive dimensions captured by reveiwing field notes and memos when analysing the data.

This paper only describes the experiences of IENs which limits the ability to evaluate the accurateness of some of their comments. Targeted interviews with key stakeholders (regulatory body and employer) have been completed and after analysis we will be able to report on discrepancies between the IENs experiences and those of the regulatory and recruiting institutions. The study was essentially a preliminary small scale (pilot) study with a view to conduct a larger national study; therefore the findings are not generalizable but important principles can be elicited. It is also recognized that during the progression of this study a number of provincial and national strategies were initiated such as the *Pan Canadian Framework for the Recognition of Foreign Credentia*ls [35] in Canada during 2009/2010, however our study pre-dates the implementation of these strategies, therefore unfortunately participants in this study were not their beneficiaries. Nevertheless, the transitioning of current and future IENs will likely be enhanced with these welcome initiatives.

### Discussion of the findings

Overall, this study revealed that IENs recruited during 2008-2009 were a group of well-educated nurses from a variety of countries who often had other experiences of working in foreign countries and gaining credentials. Many IENs stated that their expectations, largely with respect to professional role, were unmet and that they felt disillusioned about the regulatory requirements to practice as RNs and the ability for them to practice in the specific area of their expertise. The majority of the IENs in this study judged their experiences with reception and support upon arrival as negative. Much of their negativity seemed to stem from faulty expectations related to their nursing position level and specialty, but much was also related to the discrepancy of their need for a place to live yet being provided one which was thought to be unsuitable (primarily in location) and not fit for a health professional.

Significantly, many of the IENs had worked in other countries besides Canada and their country of origin (around 50% of the IENs in the study had multiple registrations) which may suggest that this group of nurses are particularly adventurous, but also that their prior experience of reception and support in other nation states would enable them to be quite capable of appraising the relative support provided in Canada.

There do seem to be some significant issues surrounding the recruitment process, especially in relation to provision of and access to information. Recruitment agencies were it appears failing to provide accurate and relevant information. It is not clear the extent to which the health authority was in a position to influence the delivery of this information in the home countries of the migrating IENs. It does seem that some IENs acted with a degree of naivety and perhaps ought to have asked more probing questions at the time of recruitment. Regardless, recruiting IENs on short contracts results in a loss of their permanent jobs, uprooting of their families and lives led with a lot of uncertainties.

### Policy and Practice Recommendations

Three major policy and practice recommendations were developed upon reflection of this study's findings:

• Clear communication and transparent expectations need to be delivered from the employing health authority to the recruiting agencies, to enable accurate information of the credentialing process and orientation structure. Nurses should not be given any expectation of specific work specialties unless this (via occupancy of the position and competency of the IEN) can be determined without doubt prior to their arrival. It should be clearly explained to IENs that starting their work within a generalist area (general medicine-surgical unit) provides the best opportunity for them to integrate successfully.

• Orientation/bridging/retraining programs need to consider findings from this and other studies (for an example see reference 24) which indicate that IENs require extensive orientation (up to 6 months) and that which considers wider social and economic dimensions of integration into a new ethno-cultural context. While there are some on-line courses on Canadian health care, culture and context available through Canadian institutions (e.g. University of Toronto; http://www.iehpcanada.utoronto.ca/index.html) for orientating Internationally-Educated Health Professionals either prior to or after their arrival, this material should be explicitly provided to newly-recruited nurses prior to their migration. Since language and communication problems are paramount for credentialing and transitioning of many IENs, programs which assist with acquiring skills for communication within the Canadian health care System (e.g. http://www.englishforhealth.com/) should be offered at the recruitment stage.

• Nurse managers need to be provided with comprehensive training programs on cultural competency (of their employees) and empowering their units to embrace and celebrate diversity and the contributions IENs make to the health care system.

## Conclusions

Recruitment of IENs can offer significant contributions to health care provision in countries where shortages are increasing. Looking to international literature can help inform decision-makers and health care managers when hiring IENs, allowing for critical appraisal of the challenges faced and strategies used for transitioning and retaining the IENs in numerous ethno-cultural and health care contexts. The situation studied here was challenging due to significant reformation of the health care governance within the Canadian province studied, yet major issues during the recruitment stages should not be overlooked as many IENs globally will use similar methods of gaining employment.

## Competing interests

The author declares that they have no competing interests.

## Authors' contributions

The author conceived and designed the study, obtained the grant funding, oversaw preparation of the participant information and recruitment materials, presented the study to nurses, and oversaw and contributed to data collection, management and analysis. She revised the drafted manuscript critically for intellectual content and approved the final version for submission.

## Pre-publication history

The pre-publication history for this paper can be accessed here:

http://www.biomedcentral.com/1472-6955/10/14/prepub
